# Serological Response to SARS-CoV-2 Vaccine in Hemodialyzed Patients and the Association with Later COVID-19 Positivity

**DOI:** 10.3390/antib12020037

**Published:** 2023-05-24

**Authors:** Vedran Premuzic, Ranko Stevanovic, Tatjana Vilibic-Cavlek, Maja Sirovica, Sara Stalman, Maja Bogdanic, Denis Zilic, Dario Nakic, Danijela Santini Dusevic, Marina Vojkovic, Jerko Barbic, Ivan Durlen, Zeljka Grdan, Drasko Pavlovic, Boris Kudumija, Sinisa Sefer, Davor Griparic, Dunja Rogic, Marija Bubas, Krunoslav Capak, Bojan Jelakovic

**Affiliations:** 1 Department of Nephrology, Hypertension, Dialysis and Transplantation, University Hospital Center Zagreb, 10000 Zagreb, Croatia; jelakovicbojan@gmail.com; 2 School of Medicine, University of Zagreb, 10000 Zagreb, Croatia; maja.sirovica@gmail.com (M.S.); sara.stalmann@gmail.com (S.S.); draskop1311@gmail.com (D.P.); 3 Croatian Institute of Public Health, 10000 Zagreb, Croatia; rankostev@hotmail.com (R.S.); maja.bogdanic@hzjz.hr (M.B.); bubas.hzjz@gmail.com (M.B.); krunoslav.capak@hzjz.hr (K.C.); 4 Axon Lab d.o.o., 10000 Zagreb, Croatia; denis.zilic@axonlab.com; 5 Department of Nephrology and Dialysis, General Hospital Zadar, 23000 Zadar, Croatia; 9dario.nakic@gmail.com (D.N.); d.santini.dusevic@gmail.com (D.S.D.); marina.vojkovic03@gmail.com (M.V.); 6 Department of Nephrology, Dialysis and Transplantation, Clinical Hospital Center Osijek, 31000 Osijek, Croatia; jerko.barbic@mefos.hr; 7 Department of Nephrology and Dialysis, University Hospital Dubrava, 10000 Zagreb, Croatia; durlenivan@gmail.com; 8 Department of Nephrology, Clinical Hospital Varazdin, 42000 Varazdin, Croatia; zeljka.grdjan@gmail.com; 9 Policlinic for Internal Medicine and Dialysis B. Braun Avitum, 10000 Zagreb, Croatia; bkudumija@avitum.hr; 10 Department of Nephrology and Dialysis, University Clinical Hospital Centre “Sestre Milosrdnice”, 10000 Zagreb, Croatia; sinisazgb1@gmail.com; 11 Policlinic for Dialysis Fresenius Sveti Duh 2, 10000 Zagreb, Croatia; griparic.davor@gmail.com; 12 Clinical Institute for Laboratory Diagnostics, University Hospital Center Zagreb, 10000 Zagreb, Croatia; predstojnik.lab@kbc-zagreb.hr

**Keywords:** serological response, SARS-CoV-2, hemodialysis, vaccination, COVID-19

## Abstract

Background: The effectiveness of the COVID-19 vaccine may differ in hemodialysis patients. The aim of this prospective multicenter study was to determine the degree of serological response to the SARS-CoV-2 vaccine in the population of dialysis patients and its association with later SARS-CoV-2 infections. Methods: A blood sample was taken for the determination of COVID-19 serological status (IgG antibodies) in 706 dialysis patients 16 weeks after vaccination with the second dose (Pfizer-BioNTech). Results: Only 314 (44.5%) hemodialyzed patients had a satisfactory response to the COVID-19 vaccine. Eighty-two patients (11.6%) had a borderline response, while 310 patients (43.9%) had an unsatisfactory (negative) post-vaccinal antibody titer. A longer dialysis vintage had an increased odds ratio (OR) of 1.01 for the occurrence of COVID-19 positivity after vaccination. In the group of subsequently positive patients, 28 patients (13.6%) died from complications of COVID-19. We have found differences in mean survival time between patients with and without appropriate responses to vaccination in favor of patients with a satisfactory serological response. Conclusions: The results showed that the dialysis population will not have the same serological response to the vaccine as the general population. The majority of dialysis patients did not develop a severe clinical picture or die at the time of positivity for COVID-19.

## 1. Introduction

The prevalence of COVID-19 in patients with end-stage renal failure on dialysis is 3% [1]. Most of them are infected at an older age and have a history of cardiovascular diseases and diabetes [2]. A meta-analysis by Nopsopon et al. shows that the case fatality rate in dialysis patients with COVID-19 is 18% [1] while studies from different countries reported various mortality rates [3]. Research conducted in the United States of America showed that the mortality rates of COVID-19 patients on dialysis are between 28% and 32%, and those conducted in Spain are between 25% and 31%. A mortality rate of 20% has been described in the United Kingdom, 26% in Italy, and 24% to 27% in France [3].

According to a European Renal Association COVID-19 Database (ERACODA) report, patients on hemodialysis had an increased risk of death, with a 25% 28-day probability of death and 33.5% for patients who required hospitalization [4].

Given the presence of various comorbidities, an impaired immune response, and frailty in chronic patients, the effectiveness of the vaccine may differ from that of the general population [5,6]. Chronic kidney patients on dialysis are at increased risk of a more severe form of COVID-19 infection and a high mortality rate [7]. Research by Dulovic et al. shows that vaccine protection Comirnaty declined rapidly in dialysis patients 4 months after the second dose of the vaccine. As many as 19.7% of dialysis subjects were seronegative 16 weeks after the second dose of the vaccine, and 75% of them had a significant drop in antibody levels [8]. Patients on dialysis have impaired cellular and humoral immunity, which makes infections the second leading cause of death in dialysis patients after cardiovascular diseases [9]. They cause death in 20% of patients with end-stage kidney disease [10]. Failure of kidney function leads to the accumulation of toxic products, which we call uremia. Uremia causes a loss of balance between pro- and anti-inflammatory factors and pro- and anti-apoptotic factors, which causes immunosuppression. Due to the deficient response of T-lymphocytes to the stimulus, the creation of immunity after vaccination is inadequate [11].

A possible reason for the lower rate of a severe form of COVID-19 and the lower mortality rate in dialysis patients is the hypoimmune response to the infection, which is a consequence of the patient’s immunosuppression and the high vaccination coverage of the dialysis population. The aim of this prospective multicenter research was to determine the degree of serological response to the vaccine against SARS-CoV-2 in the population of dialysis patients and its association with later SARS-CoV-2 infections.

## 2. Materials and Methods

### 2.1. Patients

A total of 706 dialysis patients from eight Croatian dialysis centers were included in the study. After the participants were vaccinated with the second dose vaccination with a 21-day interval of BNT162b2 (Pfizer-BioNTech, Mainz, Germany), a blood sample was taken by using vascular access before the start of dialysis 16 weeks after vaccination for the determination of COVID-19 serological status (IgG antibodies). The control group consisted of 372 healthy individuals.

Basic demographic data on all patients, including data on their body height and weight, smoking habits, dialysis, comorbidities, and medications they are taking, was collected from the hospital information system. The use of angiotensin-converting enzyme (ACE) inhibitors or immunosuppression therapy was also collected for all patients. Values of blood urea nitrogen (BUN), creatinine, erythrocytes, platelets, hemoglobin, hematocrit, lymphocytes, white blood count, serum calcium, phosphates, cholesterol, triglycerides, and uric acid were also collected, as well as data on the COVID-19 positivity of hemodialyzed patients after vaccination and the booster dose.

### 2.2. Serological Testing

The initial serological screening was performed using a commercial automated enzyme-immunoassay based on recombinant spike glycoprotein (S) and nucleocapsid protein (N) antigens of SARS-CoV-2 (ELISA COVID-19 IgG; Vircell Microbiologists, Granada, Spain). The test is based on the reaction of SARS-CoV-2 antibodies in the sample with the antigen adsorbed on the microtiter plate. After washing off unbound immunoglobulins, an enzyme-labeled antihuman IgG binds to the antigen-antibody complex. After a new washing step, a substrate solution (tetramethylbenzidine) is added, and color will develop. After adding a stop solution (0.5 M sulphuric acid), optical densities (OD) are read with a spectrophotometer at 450/620 nm within one hour of stopping. Results were calculated and expressed as an antibody index; AI = (sample OD/cut- +off serum mean OD) × 10. Samples were considered positive if AI values were >6, borderline if values were 4–6, and negative if values were <4.

All reactive samples were further confirmed using an automated surrogate neutralizing fluorescence immunoassay (FIA; AFIAS COVID-19 nAb, Boditech Med Incorporated, Gang-won-do, Korea). The test uses a competitive immunodetection method for qualitative determination of SARS-CoV-2 surrogate neutralizing (sNT) antibodies that block the interaction between the receptor-binding domain (RBD) of the SARS-CoV-2 S glycoprotein and the ACE-2 cell surface receptor. SARS-CoV-2 sNT antibodies in the serum sample bind to the fluorescence-labeled SARS-CoV-2 RBD antigen and form a complex. The complex migrates onto the nitrocellulose matrix with immobilized ACE-2 and interferes with the binding of sNT antibodies and fluorescence-labeled RBD. If more sNT antibodies are present in the sample, fewer detection antigens are accumulated, resulting in a lower fluorescence signal. Results were calculated based on inhibition rate (%) and interpreted as follows: cut-off index (COI; %) <30 negative; ≥30 positive.

### 2.3. Statistical Analysis

The normality of the data distribution was tested using the Kolmogorov-Smirnov test. Preliminary analyses were performed to ensure no violation of the assumptions of normality, linearity, and homoscedasticity. Categorical data were expressed as numbers and frequencies. Correlations were obtained using Pearson’s test for normally distributed variables and Spearman rank correlation for non-normally distributed variables. Normally distributed variables were presented as means ± standard deviations, and the Student’s *t*-test for independent samples was used for comparisons between the two groups. Non-normally distributed data were presented as a median and interquartile range, and the Mann-Whitney U-test was used for the comparison between the two groups. Baseline-to-follow-up comparisons were done using the Student’s *t*-test for paired samples and the Wilcoxon test. Categorical variables were compared using the χ^2^-test. Multiple linear regression and multiple nominal regression were used to explore the influence of different variables on antibody titer, while logistic regression was used for categorical dependent variables. A *p*-value < 0.05 (two-sided tests) was considered significant. Survival probability curves were generated by means of the Kaplan-Meier method and analyzed by the log-rank (Mantel-Cox) test. Hazard ratios (HRs) and 95% CIs were estimated by the Cox proportional hazards regression method (Cox regression). Statistical analyses were performed using SPSS version 23.0 (IBM Corp., Armonk, NY, USA).

## 3. Results

Out of the total number of included vaccinated patients on dialysis, only 314 patients (44.5%) had a satisfactory response to the COVID-19 vaccine (positive SARS-CoV-2 IgG antibodies). Eighty-two patients (11.6%) had a borderline response, and even 310 patients (43.9%) had an unsatisfactory (negative) antibody titer to the vaccine. The total percentage of borderline and negative patients was 55.5%. By comparing the dialysis patients with satisfactory and non-satisfactory serological responses (borderline and no response patients), we have found that patients with non-satisfactory serological responses were significantly older, while other differences in dialysis-related parameters, medications, or laboratory values between groups were not found (Table 1). We have not found any adverse effects of vaccination in hemodialysis patients after injection or aggravation of their primary disease during the following weeks.

**Figure 1:**
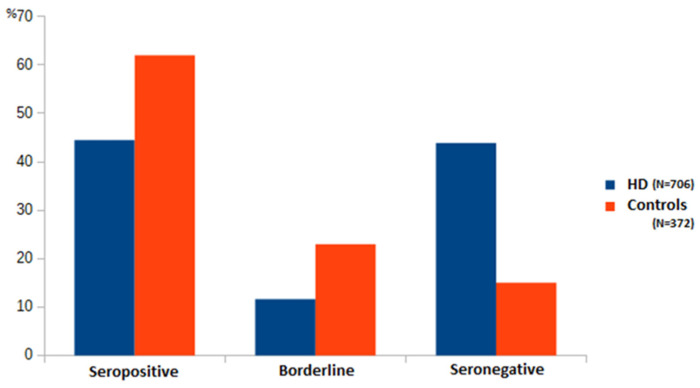
Percentage of patients with different serologic responses between hemodialyzed patients and the control group of subjects.

By comparing the serological response of dialysis patients with the control group of healthy subjects, it is evident that the healthy had a better response (62% positive, 23% borderline response, and only 15% without an adequate serological response) (Figure 1). The total percentage of borderline and negative healthy subjects was 38%. Although the control group was sex-matched with the group of dialyzed patients, the subjects in the control group were significantly younger (*p* < 0.05).

Univariate analysis found a significant negative association of antibody titer with higher age (*p* < 0.01), higher serum creatinine levels (*p* < 0.01), and the duration of dialysis (*p* = 0.04). No association of antibody levels with other dialysis parameters, chronic drug therapy, or patient comorbidities was found.

In the linear regression analysis, we have not found a statistically significant association of age, dialysis-related parameters, medications, or laboratory values with antibody levels.

The results of the logistic regression analysis showed a risk ratio for a lower antibody level after vaccination of 1.1 for a higher age.

By comparing the dialysis patients with and without COVID-19 positivity after vaccination, we have found that patients with COVID-19 positivity had significantly higher serum creatinine levels and lower antibody titers.

On logistic regression, longer dialysis vintages had an increased OR of 1.013 (95% CI = 1.002–1.025) for the occurrence of COVID-19 positivity after vaccination (Table 2).

As many as 71% of patients did not develop COVID-19 seropositivity after vaccination with two doses and a booster dose of the vaccine, with no significant difference between patients with satisfactory and non-satisfactory responses to vaccination. Of the 29% of subsequently positive patients, 28 patients (13.6%) died from complications of COVID-19. The rest of the patients had a medium-severe or mild clinical picture of COVID-19 infection. By analyzing patients who developed a mild or moderate clinical picture of COVID-19, no difference was found in the number of patients depending on the serological response to the vaccine. A total of 28 vaccinated patients died from COVID-19 infection; twenty-one did not have an appropriate serological response to the vaccine, and seven had an appropriate response to the vaccine (*p* = 0.02). A longer dialysis vintage was associated with higher mortality in the whole group (HR 1.01 [1.00, 1.02], respectively) (Table 3).

**Figure 2:**
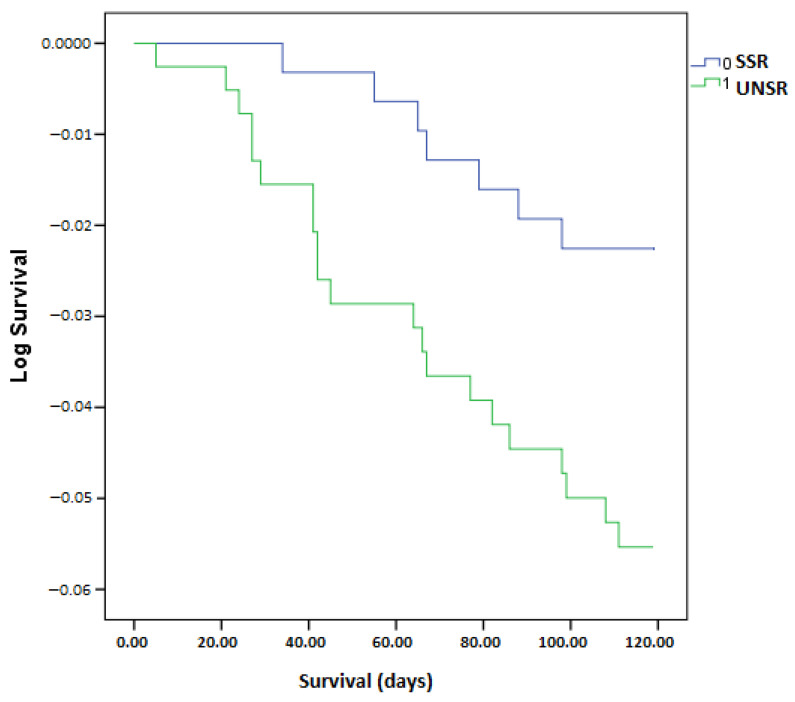
Differences in survival between patients with and without satisfactory serologic response (118.8 days; 95% CI = 117.9–119.7 vs. 116.6 days; 95% CI = 115.0–118.2 days; *p* = 0.03): SSR = satisfactory serologic response; UNSR = unsatisfactory serologic response.

We have found differences in mean survival time between patients with and without appropriate responses to vaccination in favor of patients with a satisfactory serological response (Figure 2).

## 4. Discussion

The results of this study showed that the serological response to the COVID-19 vaccine in patients in a chronic dialysis program was absent or unsatisfactory in more than half of those vaccinated and significantly different when compared to healthy controls. Antibody levels were significantly negatively associated with older age and dialysis vintage.

There are several possible reasons why dialysis patients had a poor response to the COVID-19 vaccine. When the effectiveness of the vaccine was examined for the general population, it was described that the antibody titer decreased over time [12]. Similar data are available for patients with chronic diseases [13]. The results of previous studies on patients with chronic diseases showed that the protection against the vaccine decreased rapidly a few weeks after vaccination. For oncology patients, the effectiveness of the vaccine largely depended on the time of completion of systemic therapy (chemotherapy, hormone therapy) and ranged from 58% to 85% [5,6]. If the therapy was completed 6 months before receiving the first dose of the vaccine, the effectiveness was higher and amounts to 85%, while in patients whose therapy was completed less than 6 months before the first dose of the vaccine, the effectiveness was lower and amounts to only 58% [5]. Research by Monin et al. also showed the effectiveness of the vaccine in the population of oncological and hemato-oncological patients, and it was evident that in this population, the immune response after the first dose of the vaccine was extremely weak. Seroconversion was 38% in patients with solid tumors and 18% in hemato-oncological patients compared to healthy controls, where seroconversion was present in 98% of cases. After the second dose, seroconversion increased to 95% in the population with solid tumors but to only 60% in haemato-oncology patients [6].

Similar reports were published regarding rheumatological patients, which were attributed to immunosuppression drugs [13]. Research by Furer et al. indicated a lower seropositivity rate 2–6 weeks after the second dose of the Comirnaty vaccine in patients suffering from autoimmune inflammatory rheumatic diseases, and it was 86% in this population, compared to healthy controls, where the seropositivity rate was 100%. This was attributed to the drugs taken by rheumatological patients, which have the common goal of immunosuppression and calming inflammation: glucocorticoids, mycophenolate mofetil, rituximab, and abatacept [13]. Similar to the present paper, the study by Pellicano et al. conducted on patients with systemic sclerosis demonstrated an impaired response to the COVID-19 vaccine [14].

Given that dialysis patients are a high-risk population for contracting severe forms of COVID-19, they were on the priority list for vaccination. Simon et al. reported a significantly lower antibody level three weeks after receiving the second dose of the Comirnaty vaccine in dialysis subjects compared to the control group (171 U/mL vs. 2500 U/mL) [15]. Anand et al. analyzed 2563 dialysis patients who were vaccinated with two doses of the vaccine against COVID-19. Serological testing was performed on them once a month, for the purpose of studying the long-term protection of the vaccine in hemodialysis patients. The study showed that 20.2% of patients had undetectable antibody levels after 6 months [16]. Yanay et al. also demonstrated lower antibody titers in hemodialysis patients compared to healthy controls [17]. These reports can imply a weaker antibody response in dialysis patients even after two doses of the vaccine, which disables them from neutralizing the SARS-CoV-2 virus. Nevertheless, a delayed response is possible in the case of natural infection due to the reported increased seroconversion rate in COVID-infected HD patients [18].

The results of our study show an even higher percentage of seronegative patients who were vaccinated with two doses of the vaccine against COVID-19 than in previous studies [8]. A lower antibody level after vaccination in our group of dialysis patients was significantly negatively associated with a higher age, which was also described in studies investigating the serological response to the infection in the general population [19] and with a longer dialysis vintage. Possible reasons for this association lie in the impaired immune response of dialysis patients. The longer dialysis vintage leads to a gradual decrease in cellular and humoral immunity, which is one of the reasons why infections are the second leading cause of death in this population [20]. Likewise, long-term dialysis is responsible for the deposition of uremic toxins in the tissues and the simultaneous loss of balance between pro- and anti-inflammatory factors, which consequently results in immunosuppression [21].

A number of other parameters of dialysis treatment, such as iron administration, elevated calcium, and PTH values, lead to lymphocyte dysfunction and a worse immune response to the vaccine [22]. Overloading with accumulated iron and elevated values of intracellular calcium, caused by kidney failure, causes a decrease in the function of polymorphonuclear leukocytes [21]. Inhibitory proteins such as GIP I, GIP II, and DIP also act on polymorphonuclear leukocytes [21,22]. High PTH values affect the function and metabolism of B-lymphocytes and the function of T-lymphocytes. The uremic toxin indoxyl sulfate suppresses the expression of erythropoietin [23]. EPO participates in the differentiation of dendritic cells, making them more sensitive to stimuli. Other uremic toxins such as phenylacetic acid, guanidine compounds, methylglyoxal, leptin, and resistin promote apoptosis of immune system cells or inhibit their activity [20].

The results of our study showed that most of the vaccinated patients had a mild or moderate COVID-19 disease, which is indirect proof of vaccine effectiveness in reducing the more severe forms of the disease. Due to the high risk of infection and more severe forms of COVID-19 in chronic patients, most of our patients (more than 90%) had a high vaccination rate, which contributed to their lower fatality rate, although at the time the highly contagious delta variant of the virus, which caused more severe forms of COVID-19, was predominant.

Cardiovascular diseases, diabetes, obesity, respiratory, malignant, nephrological, hepatological, and neurological diseases are risk factors both for the severe form of COVID-19 and for fatal outcomes [24]. Geng et al. in their meta-analysis reported that 36.49% of patients who died from COVID-19 had at least one chronic disease. The most common cases were hyperlipidemia and hypertension [25]. Arrhythmias, shock, acute respiratory distress syndrome, and acute heart failure occur more often in dialysis patients compared to the general population [26]. It has been shown that dialysis patients are more prone to thrombosis after COVID-19 infection, and it occurs in 18.5% of patients who have recovered from COVID-19 and only in 1.9% of those who have not [27]. In the long term, as many as 93.7% and 81% of those infected report symptoms such as general weakness or palpitations 3 months and 6 months after infection, respectively [28].

The mortality of patients with COVID-19 in the chronic dialysis program is described in European countries as 20–32% [1,3]. On the other hand, in Croatia, a very small number of dialysis patients developed a severe clinical picture, and the mortality rate was not high either [29]. According to the results of our research compared to other studies, the mortality rate in our group of patients was significantly lower (13.6%). The results of the logistic regression analysis showed an OR for the occurrence of COVID-19 positivity after vaccination and a booster dose of 0.9 for a lower antibody level after vaccination and 1.1 for older age. Although in positive patients who developed a mild or moderate clinical picture, the antibody level after vaccination did not play a significant role, in patients with a severe clinical picture or in deceased patients, there was a significantly higher proportion of patients who did not develop SARS-CoV-2 antibodies after vaccination, while a longer dialysis vintage was associated with higher mortality.

There are a few factors that may explain the phenomenon of low mortality in our group of hemodialyzed patients. First, the vaccination rate exceeded 90% of patients during the highly contagious delta variant of the virus. Second, a lower antibody level was associated with a longer dialysis vintage and therefore with a longer effect of uremia on cellular and humoral immunity and consequently on the development of immunosuppression. This long-term uremic milieu possibly produced a paradoxical phenomenon: the unsatisfactory serological response to the SARS-CoV-2 vaccine, which increased the risk for breakthrough infections, and at the same time the suppressed immune response to SARS-CoV-2 and more severe forms of COVID-19.

In this study, it was not possible to assess the antibody dynamic after COVID-19 vaccination in hemodialysis patients since only one serum sample (taken 16 weeks after the second dose) was available, which is one of the study’s limitations. Studies that analyzed the antibody response showed a rapid decline in antibody titers after vaccination. An Israeli study included hemodialysis patients who received at least three doses of the vaccine. The third vaccine dose induced a significant increase in IgG anti-S titers, followed by a decline over 4–5 months [30]. Similarly, in a prospective cohort study conducted in Virginia, USA, 87.88% of hemodialysis patients were SARS-CoV-2 IgG positive at baseline. Patient antibody levels decreased at a monthly average adjusted rate of 31%, and at six months after vaccination, 40% of patients had either borderline or negative IgG antibodies [31]. In addition, a study from Portugal showed that elderly patients developed a lower immune response, and the levels of anti-spike IgG antibodies declined faster than in younger patients [32].

To the best of our knowledge, this is the first study to analyze the serological response in this large number of hemodialyzed patients and its associations with later COVID-19 positivity and mortality. Our results showed that the vaccination is effective in the prevention of more severe forms of COVID-19 in these chronic patients, but the serological response is still significantly lower when compared to healthy subjects [33,34]. Interestingly, our results did not show any differences in serological response or later COVID-19 infections when patients received a booster dose of the vaccine.

This study has some other limitations that need to be addressed. We have tested only the humoral (antibodies) and not the cellular (T-cell) immune response, which probably plays a role in the protection from COVID-19. A study by Le Bert et al. [35] found CD4 and CD8 T cells possessed long-lasting memory and cross-reactivity to the N protein of SARS-CoV-2 in patients who recovered from SARS as well as in patients with no history of SARS or COVID-19. This analysis of the cellular immune response provides additional information regarding vaccination efficacy and, consequently, the association with more severe forms of COVID-19 and clinical outcomes in these patients. The importance of cellular immune response was also found in the study by Cassaniti et al. [36] which suggested that a long-term SARS-CoV-2 T-cell response might accompany a waning humoral response.

In addition, our study is limited by the relatively small sample size of patients, who were not matched by age or sex, and it represents the results from one country. Therefore, a study on a large number of patients is needed to confirm our observations.

## 5. Conclusions

The research results confirmed the assumption that our dialysis population, due to immunosuppression from long-term dialysis and uremia, will not have the same serological response to the vaccine as the general population. At the same time, this is the likely reason why the majority of dialysis patients did not develop a severe clinical form or die at the time of positivity for COVID-19.

## Figures and Tables

**Table 1:** Differences between patients with and without satisfactory serological response.

Parameter	Satisfactory Serological Response (N = 314)	Non-Satisfactory Serological Response (N = 392)	*p*
Age (years)	64 (34–82)	67 (39–86)	<0.01
BMI (kg/m^2^)	25.7 ± 5.7	25.1 ± 4.9	0.13
Male sex N (%)	183 (58.2)	237 (60.4)	0.55 *
Dialysis vintage (months)	57.4 ± 14.2	61.2 ± 15.3	0.43
Duration of hypertension (months)	143 (34–214)	141 (32–209)	0.89
Diabetes (%)	107 (34.1)	122 (31.1)	0.51 *
RAAS inhibitors N (%)	143 (45.5)	175 (44.6)	0.81 *
Immunosuppressive drugs (CNI, MMF, steroids) N (%)	87 (27.7)	90 (22.9)	0.15 *
Residual diuresis (mL)	722 ± 89.3	701 ± 88.4	0.75
BUN (mmol/L)	22.1 ± 5.7	21.15 ± 6.7	0.06
Serum creatinine (µmol/L)	788 (287–1122)	744 (265–1087)	0.21
Thrombocytes (×10^9^/L)	208 (152–244)	193 (139–231)	0.81
Hemoglobin (g/L)	118.2 ± 28.2	112.3 ± 26.9	0.22
White blood count (×10^9^/L)	5.9 ± 2.7	6.3 ± 2.9	0.37
Lymphocytes (%)	22.8 ± 5.1	21.5 ± 4.9	0.18
Serum calcium (mmol/L)	2.3 ± 0.8	2.2 ± 0.7	0.55
Phosphates (mmol/L)	2.8 ± 0.9	1.7 ± 0.4	0.25
Cholesterol (mmol/L)	3.6 ± 1.1	3.7 ± 1.2	0.35
Uric acid (µmol/L)	358 (302–412)	365 (313–422)	0.24

BMI = body mass index; RAAS = renin-angiotensin-aldosterone system; CNI = calcineurin inhibitors; MMF = mycophenolate mofetil acid; EPO = erythropoietin; BUN = blood urea nitrogen; results are shown as mean +/− SD or median (interquartile range); * χ2-test.

**Table 2:** Factors associated with COVID-19 positivity after vaccination.

Parameter	B	S.E.	Wald	*p*	Exp (B)	95% CI for Exp (B)
Age (years)	0.130	0.088	2.169	0.14	1.138	0.958–1.353
Antibody level (AU/mL)	−0.038	0.081	0.224	0.64	0.962	0.821–1.128
Male sex	−0.085	0.053	2.575	0.11	0.919	0.828–1.019
Duration of hypertension (months)	0.006	0.005	1.298	0.25	1.006	0.996–1.015
Dialysis vintage (months)	0.013	0.006	5.430	0.02	1.013	1.002–1.025
Diabetes	2.653	1.934	1.882	0.17	1.196	0.321–1.982
Serum creatinine (µmol/L)	−0.006	0.003	3.821	0.51	0.994	0.987–1.000

**Table 3:** Factors associated with mortality.

Parameter	Cox Regression Analysis	
	Adjusted HR	*p*
Age (years)	1.27	0.24
Antibody index (AI)	0.97	0.74
Male sex	0.92	0.73
Duration of hypertension (months)	1.00	0.25
Dialysis vintage (months)	1.01	0.02
Diabetes	14.19	0.17
Serum creatinine (µmol/L)	0.99	0.18

HR = hazard ratio.

## Data Availability

Not applicable.
